# Sesamin Protects Against Polystyrene Microplastics-Induced Lung Injury via Attenuating Bcl2-Mediated Apoptosis

**DOI:** 10.3390/antiox15030279

**Published:** 2026-02-24

**Authors:** Yadong Zhang, Zhenao Zhang, Huanting Pei, Chongyue Zhang, Xiaolong Zhang, Simeng Qiao, Siqi Zhu, Ziyi Wang, Jingyi Ren, Yuxia Ma

**Affiliations:** Hebei Key Laboratory of Environment and Human Health, Department of Nutrition and Food Hygiene, School of Public Health, Hebei Medical University, Shijiazhuang 050017, China; yadong_z@163.com (Y.Z.); z2236232@163.com (Z.Z.); huanting_p@163.com (H.P.); chongyue_zhang@163.com (C.Z.); zxl010720@163.com (X.Z.); simeng_qiao@163.com (S.Q.); zhus_q@163.com (S.Z.); solitude1016@163.com (Z.W.); jren@hebmu.edu.cn (J.R.)

**Keywords:** sesamin, polystyrene microplastics, lung injury, apoptosis, Bcl2

## Abstract

Studies show microplastics (MPs) impair lung function directly and indirectly, yet effective solutions are lacking. In light of this, sesamin (Ses), a natural lignan-like compound with diverse pharmacological properties, may offer protection. The study aims to investigate whether Ses pretreatment can mitigate MPs-induced lung damage and to elucidate the underlying mechanisms. Male C57BL/6 mice received MPs (10,000 μg/L) in drinking water, with varying Ses doses gavaged daily for 28 days. Computational pharmacology and in vivo/in vitro experiments, including histology, immunofluorescence, and western blot, were used to elucidate Ses’s protective mechanisms. In vivo experiments showed Ses can alleviate MPs-induced histopathological alterations, inflammatory responses, and oxidative stress in lung tissue. Computational pharmacology suggested that the protective mechanism of Ses may be associated with the apoptotic signaling pathway, with Bcl2 as its potential target. Both in vivo and in vitro studies demonstrated that Ses significantly upregulates Bcl2 expression while downregulating Bax and Casp3. Notably, a Bcl2 inhibitor substantially attenuated Ses’s protective effects. Our research suggests that Ses can mitigate MPs-induced lung injury by modulating the apoptotic signaling pathway, with Bcl2 identified as a key target. Dietary supplementation may represent a promising intervention strategy for preventing and managing food safety risks associated with MPs.

## 1. Introduction

Since the onset of mass manufacturing in the 1950s, plastic products have permeated every aspect of human life [1]. Between 1950 and 2015, global plastic waste generation reached approximately 6.6 billion tons [2]. With annual global production at 400 million tons [3], an estimated 11 billion metric tons of plastics are projected to accumulate by 2025 [4]. Microplastics (MPs), characterized by their size ranging from 0.1 μm to 5 mm, fragment into smaller sizes via weathering and sunlight exposure [5,6]. Ingestion is one of the predominant ways MPs enter into the human body [7]. Previous studies have shown the existence of MPs in various food products, such as bottled water, honey, beer, and canned fish [8]. A recent study found that multiple types of MPs are present in various human biological samples, such as lung tissue, liver tissue, breast milk, feces, placenta, and blood [9]. These findings underscore MPs as a significant food contaminant, posing substantial health risks.

MPs ingested by living organisms enter the digestive tract, where particles ≤ 130 µm can be translocated via persorption, a rare mechanical process occurring through transient gaps at villus desquamation sites. Particles < 20 µm are internalized via phagocytosis, enabling entry into the circulatory system [10,11]. Significantly, smaller MPs can migrate from the gastrointestinal tract into systemic circulation, exhibiting greater accumulation in the lungs in comparison to larger particles [12]. The lung is thus considered an important target organ of MPs toxicity. It has been shown that the mechanism underlying MPs inhalation-induced lung injury in mice may be associated with multiple pathways, such as apoptosis, oxidative stress, and inflammation [13]. However, the mechanisms underlying the activation of these pathways by MPs exposure remain to be elucidated. Moreover, the majority of studies have concentrated on the detrimental health effects associated with inhalation exposure, while the effects of intake exposure on the human body remain insufficiently explored. Given the unavoidable exposure to plastic products in daily life, developing effective intervention strategies to mitigate the negative effects of MPs on lung health is necessary.

Sesamin (Ses). a lipid-soluble lignan originating from sesame seeds, possesses antioxidant, anti-inflammatory, and anti-apoptotic properties, as supported by extensive pharmacological studies [14,15,16]. A prior study has shown that Ses can mitigate LPS-induced acute kidney injury in a dose-dependent manner by attenuating renal oxidative stress, inflammation, and apoptosis [17]. Ren et al. [18] found that Ses has the potential to attenuate lung injury triggered by fine particulate matter in rats by inhibiting apoptosis and autophagy. During the mechanism by which Ses exerts its anti-apoptotic effects, Bcl2 likely serves as a critical regulatory node for anti-apoptotic activity. However, to date, there is a notable paucity of studies investigating the potential of Ses to mitigate MPs-induced lung injury and to elucidate its underlying mechanism of action.

In summary, we propose that Ses may exert a protective effect against lung injury induced by MPs. In this study, mice received MPs in drinking water, with varying concentrations of Ses gavaged daily. Transcriptomic analysis and network pharmacology methodologies were employed to investigate the underlying mechanisms through which Ses confers its protective effects. Further in vivo and in vitro experiments were used to verify the mechanisms.

## 2. Materials and Methods

### 2.1. Bioinformatics Analysis

The gene expression dataset GSE238065 was obtained from the Gene Expression Omnibus (GEO, https://www.ncbi.nlm.nih.gov/geo/, accessed on 10 June 2024) database. The dataset comprised RNA expression profiles from four normal lung epithelial cell samples and four lung epithelial cell samples exposed to 30 k nylon fibers. Differentially expressed genes (DEGs) analysis was conducted utilizing DESeq2 (version 1.40.2) in R. |log2FoldChange| > 0.5 and adjust *p* < 0.05 were set as the cutoff criteria for screening DEGs. Principal component analysis (PCA) was conducted employing the FactoMineR package. The volcano plot and heatmap were subsequently constructed using the “ggplot2” and “pheatmap” packages, respectively. Pathway enrichment analysis for the full gene list was performed with WikiPathways using gseWP. The “clusterProfiler” package in the R software was employed to conduct Kyoto Encyclopedia of Genes and Genomes (KEGG) enrichment analysis, Gene Ontology (GO) enrichment analysis, and Gene Set Enrichment Analysis (GSEA). The genes related to apoptosis were sourced from a prior study [19]. Subsequently, we utilized the GSVA package in R software to perform single-sample Gene Set Enrichment Analysis (ssGSEA) for calculating the gene set enrichment score. The results of GO enrichment analysis denoted biochemical processes (BP), cell components (CC) and molecular functions (MF). A *p*-value less than 0.05 was adopted for enrichment analysis, and the results were visualized through an online platform (https://www.bioinformatics.com.cn/, accessed on 18 June 2024).

### 2.2. Computational Pharmacology-Based Prediction

The candidate targets of Ses were predicted by utilizing the Comparative Toxicogenomics Database (CTD, https://ctdbase.org/, accessed on 4 June 2024), Bioinformatics Analysis Tool for Molecular mechanism of Traditional Chinese Medicine (BATMAN-TCM, http://bionet.ncpsb.org.cn/batman-tcm/#/home, accessed on 4 June 2024), Swiss Target Prediction (http://swisstargetprediction.ch/, accessed on 4 June 2024) and Similarity Ensemble Approach (SEA, https://sea.bkslab.org/, accessed on 4 June 2024). Subsequently, the targets were obtained utilizing the “VennDiagram” package (1.6.20) in the R software. The STRING database (https://cn.string-db.org/, accessed on 6 March 2025) was used to construct the protein–protein interaction (PPI) network. Afterward, the PPI network was incorporated into Cytoscape software (version 3.9.1). Various topological methods in the CytoHubba plugin, including MCC, EPC, Betweenness, Stress, MNC, Degree, EcCentricity, Closness, and Radiality, were used to further screen core proteins.

### 2.3. Random Walk with Restart (RWR)

The Random Walk with Restart (RWR) technique is a well-established method for network structure analysis. By incorporating a probability of restarting the random walk at each step, RWR effectively accounts for both local and global topological features of the network, thereby facilitating the identification of relevant or significant nodes within the network. The Personalized PageRank (PPR) algorithm functions as a centrality measure within a graph, where the computation of each node’s score is directly related to the number of edges incident to the nodes. In this study, all analyses were performed using Python (version 3.9) with the NetworkX library (version 3.2.1) for the construction and manipulation of graphs, supplemented by the built-in random module for stochastic processes. The PPI network was constructed from a whitespace-delimited text file containing undirected edges (one pair of node identifiers per line). A starting node was randomly selected using random.choice from the graph’s nodes. Each source node was initialized with 10,000 random walks, with a maximum of 100 steps per walk, and a restart probability set at 0.1. Genes were subsequently ranked in descending order.

### 2.4. Reagents

Ses (≥98% by HPLC) was obtained from Aladdin Company (Shanghai, China). Polystyrene Microspheres (PS-MPs) were obtained from BaseLine ChromTech Research Centre (Tianjin, China) in a distilled water carrier. Carboxymethylcellulose (CMC), a glutathione (GSH) assay kit, and a total antioxidant capacity (T-AOC) assay kit were purchased from Solarbio Science & Technology Co., Ltd. (Beijing, China). ELISA kits for interleukin-1β (IL-1β), IL-6 and tumor necrosis factor-α (TNF-α) were acquired from Mlbio (Shanghai, China). A superoxide dismutase (SOD) assay kit and malondialdehyde (MDA) assay kit were purchased from Nanjing Jiancheng Bioengineering Institute (Nanjing, China). The antibody against Bcl2 was purchased from Arigo Biolaboratories (Taiwan, China). The antibody against Bax in vivo was purchased from HuaAn Biotechnology Co. (Hangzhou, Zhejiang, China). The antibody against Casp3 in vivo was purchased from Cell Signaling Technologies (Danvers, MA, USA). Secondary antibodies were purchased from Abways Technology (Shanghai, China). The hematoxylin-eosin (H&E) kit was purchased from Servicebio Technology Co., Ltd. (Wuhan, China). An Apoptosis Detection Kit for Fluorescent Labeled TUNEL Cells was purchased from Biyuntian Biotechnology (Shanghai, China). MLE-12 cells, a mouse lung epithelial cell line, were purchased from Saibaikang Biotechnology Co., Ltd. (iCell-m036, Shanghai, China). DMEM/F12 medium, foetal bovine serum (FBS), trypsin, phosphate-buffered saline (PBS) and Cell Counting Kit-8 (CCK-8) were purchased from ZETA LIFE Inc. (San Francisco, CA, USA). Venetoclax (VEN), a highly potent Bcl2 inhibitor, was purchased from MCE (Shanghai, China). Antibodies against Bax and Casp3 in vitro were purchased from Servicebio Technology Co., Ltd. (Wuhan, China).

### 2.5. Preparation of Ses and PS-MPs

For the gavage, Ses was dissolved in a 0.5% CMC solution to prepare suspensions with concentrations of 2.5 mg/mL for the low-dose group, 5 mg/mL for the middle-dose group, and 10 mg/mL for the high-dose group. In total, 1 μm PS-MPs (sphere, dissolved in distilled water at concentrations of 10,000 μg/L) were used for the in vivo toxicological experiments.

### 2.6. Animal Experiments

Thirty 5-week-old male C57 BL/6 mice were purchased from Weitong Lihua Experimental Animal Technology Co., Ltd. (Beijing, China). They were housed under specific pathogen-free (SPF) conditions at 20–24 °C with a 12 h light/12 h dark cycle for 1 week before experiments. The animal experiments were conducted according to the Laboratory Animals’ Guiding Principles and sanctified by the Animal Experimentation Ethics Committee of Hebei Medical University (IACUC-Hebmu-2024052, 2 May 2024). Mice were randomly assigned to 5 groups (*n* = 6 per group): (i) control group (control), (ii) polystyrene microplastics exposure group (PS-MPs), (iii) low-dose Ses group (25 mg/kg, Ses-L), (iv) medium-dose Ses group (50 mg/kg, Ses-M), and (v) high-dose Ses group (100 mg/kg, Ses-H). Ses at a dosage of 10 mL/kg body weight was administered orally to the mice in the Ses-treated groups. In contrast, 0.5% CMC at the same dosage was administered orally to the control and PS-MPs exposure groups. The treatments were administered daily for 28 days. Throughout the study, the control group was provided with distilled water, while the other four groups received aqueous solutions of PS-MPs (10,000 μg/L). After 4 weeks, the mice were sacrificed, and their lungs were collected and weighed. Each lung was then divided into two segments: one was snap-frozen in liquid nitrogen for subsequent analysis, and the other was fixed in 4% paraformaldehyde for histological evaluation.

### 2.7. Assessment of Lung Tissue Edema

The lower lobe of the right lung of each mouse was excised and cleaned three times with PBS. Following the determination of the wet weight (W) of the lungs, the lung tissue was encased in aluminum foil and subjected to a drying process at 65 °C for 72 h, after which the dry weight (D) was measured. Eventually, the lung water content was calculated in accordance with the following formula: lung water content = (W − D)/W × 100%. This revealed the level of water content of the lung tissues.

### 2.8. Histological Analysis

Lung tissues were fixed in 4% paraformaldehyde and processed for paraffin embedding following standard histological procedures. Tissue blocks were sectioned at a thickness of 4 μm and stained with H&E in accordance with kit instructions. The severity of lung injury was assessed utilizing a previously established scoring system [20]. A TUNEL assay was conducted for the assessment of apoptosis in lung tissue. The tissue sections underwent deparaffinization and rehydration, followed by the application of a proteinase K working solution to facilitate antigen retrieval and equilibrium at ambient room temperature. Subsequently, the sections were treated with a mixture of TDT enzyme, dUTP, and buffer in a ratio of 1:5:50, followed by incubation at 37 °C. Then, DAPI was used to counterstain the nucleus.

### 2.9. Cell Cultures and Treatments

MLE-12 cells were cultured in DMEM supplemented with 10% FBS, 1% penicillin, and 1% streptomycin at 37 °C in a humidified atmosphere with 5% CO_2_. In the experiment, MLE-12 cells were divided into four groups: (i) control group, (ii) PS-MPs exposure group (250 μg/mL), (iii) PS-MPs (250 μg/mL) + Ses (20 μM), and (iv) PS-MPs (250 μg/mL) + Ses (20 μM) + VEN (1 μM).

### 2.10. Cell Viability Assay

To evaluate cell viability in this study, CCK-8 kits were applied. The MLE-12 cell line was seeded into 96-well plates at a density of 8 × 10^3^ cells per well for 12 h (100 μL per hole). Subsequently, the cells were treated with varying concentrations of Ses, VEN, or PS-MPs for a duration of 24 h. Following treatment, the CCK-8 solution was mixed into the culture medium at a 1:10 dilution ratio for each well. The cells underwent further incubation at 37 °C in a 5% CO_2_ environment for one more hour.

Cell viability was evaluated by measuring absorbance at 450 nm with an enzyme-labeled instrument (Spectramax M2e Multi-Mode Microplate Reader; Molecular Devices, Sunnyvale, CA, USA). The microplate was automatically shaken for 5 s before each reading.

### 2.11. Measurement of Oxidative Stress and Inflammation

The levels of various oxidative stress markers, including SOD, GSH, MDA, and T-AOC, were assessed in lung tissues and MLE-12 cells using specific assay kits. The concentrations of inflammatory cytokines, including IL-6, IL-1β, and TNF-α in lung tissues were measured by an enzyme-linked immunosorbent assay in consonance with manufacturer protocols.

### 2.12. Flow Cytometric Analysis of Reactive Oxygen Species (ROS)

The lungs of mice were dissected in ice-cold PBS. The lung tissue underwent digestion with trypsin at 37 °C for a duration of 20 min. Following the completion of digestion, the sediment was subjected to filtration using a 70 μm filter screen and subsequently centrifuged at 1200 rpm for a duration of 10 min. Red blood cell lysis buffer was then added after discarding the supernatant. The cell suspension was gathered and subsequently spun at 450× *g* for 10 min. Then, 1 mL of PBS was added to resuspend cells, followed by discarding the supernatants. The cells were incubated with DCFH-DA (Nanjing Jiancheng Bioengineering Institute, Nanjing, China) for 30 min at 37 °C in the dark. Afterwards, the cells underwent 2 PBS rinses and were analyzed using flow cytometric analysis.

### 2.13. Western Blot

The lung tissue or cells were lysed using the combination of RIPA buffer and protease inhibitors, and the protein content was quantified employing the Lowry method. SDS-PAGE (8–12%) was utilized to separate equivalent quantities of protein lysates, which were subsequently electrotransferred onto PVDF membranes. The membranes were subsequently blocked in 5% skim milk for 2 h and then cut into bands and incubated at 4 °C overnight with specific primary antibodies. The following day, the blots underwent 3 washes with TBST for 10 min each time and were incubated with the suitable secondary antibody for 1 h at room temperature. Thereafter, the strips were washed again with TBST three times. Eventually, antibody binding was detected utilizing an enhanced chemiluminescence solution and subsequently analyzed in a semiquantitative manner. The levels of protein expression were normalized to β-actin. The results were then expressed as a ratio relative to the control group, which was designated as 1.

### 2.14. Molecular Docking

The two-dimensional molecular structure of Ses (ligand) was obtained from the PubChem database (https://pubchem.ncbi.nlm.nih.gov/, accessed on 8 March 2025), while the AlphaFold structure of Bcl2 (receptor) was sourced from the UniProt database (https://www.uniprot.org/, accessed on 8 March 2025). The ligand’s two-dimensional structure was converted into a three-dimensional structure through using ChemBio3D Ultra (version 21.0.0.28), and energy optimization was performed using the molecular mechanics (MM2) force field. PyMOL software (version 2.5) was employed to remove small-molecule ligands and water molecules. Hydrogen atoms and charges were added to both ligand and receptor files using AutoDock Tools (version 1.5.7), and the files were exported in PDBQT format. Molecular docking simulations were conducted with AutoDock Vina (version 1.1.2). Finally, the docking results were visualized using PyMOL software.

### 2.15. Statistical Analyses

The SPSS 26.0 software and the GraphPad Prism 8.0.2 software were utilized to perform statistical analyses. Data were shown as the mean ± standard error of the mean (SEM) and were analyzed with one-way analysis of variance (ANOVA). *p* < 0.05 was considered statistically significant.

## 3. Results

### 3.1. Identification of DEGs in Transcriptome Dataset

The flowchart of the study is shown in Figure S1. The outcomes of the PCA (Figure S2A) demonstrated a clear separation between control and MPs exposure samples. PCA1 and PCA2 stood for 84.66% and 4.99% of the total variance, respectively. Differential expression analysis was conducted on the dataset to identify DEGs. A volcano plot was used to visualize the screening results (Figure S2B). A total of 5611 DEGs were identified, meeting the set threshold (|log2FoldChange| > 0.5 and adjust *p* < 0.05). Of these, 3097 genes were upregulated, and 2514 genes were downregulated. Afterwards, a heatmap of DEGs was exhibited in Figure S2C. Cluster analysis elucidated the expression patterns of upregulated and downregulated genes across diverse samples, with orange representing upregulation and green indicating downregulation.

### 3.2. Enrichment Analyses Revealed Changed Pathways Induced by MPs Exposure

**Figure 1:**
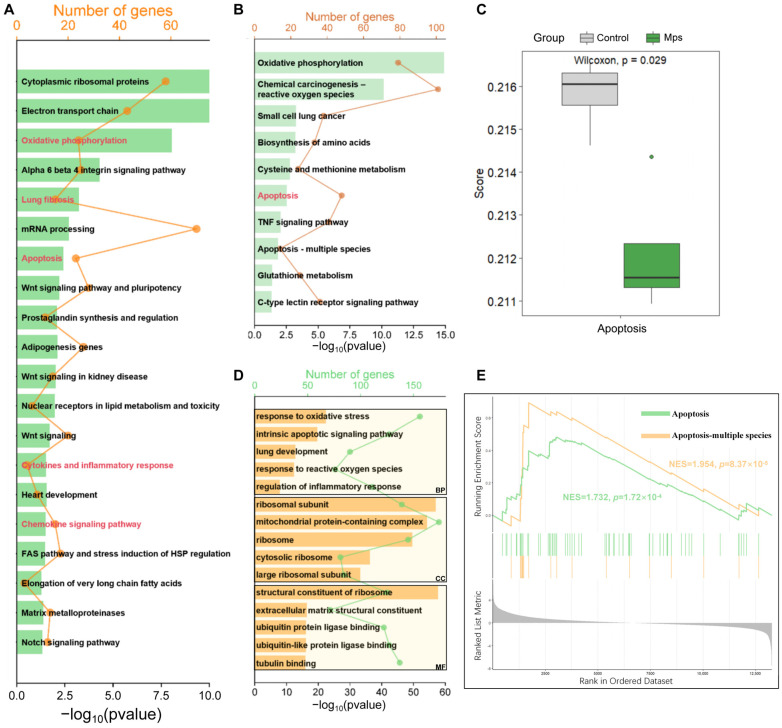
Enrichment analyses of genes. (**A**) Wikipathways enrichment analysis of full gene list. (**B**) KEGG enrichment analysis of DEGs. (**C**) ssGSEA analysis on apoptosis between the control group and the MPs exposure group. (**D**) GO enrichment analysis of DEGs. (**E**) GSEA analysis of whole genes.

The Wikipathways enrichment analysis in the study encompassed all genes and incorporated specific weak effects attributable to exposure to MPs, which did not achieve statistical significance. The application of KEGG and GO enrichment analyses was employed to elucidate the functions of DEGs. The Wikipathways enrichment analysis included 20 items. As delineated in Figure 1A, the Wikipathways enrichment analysis results indicated that the genes were significantly enriched in the oxidative phosphorylation, lung fibrosis, apoptosis, cytokines and inflammatory response, and chemokine signaling pathway. KEGG enrichment analysis of DEGs identified 97 significantly enriched pathways. As shown in Figure 1B, similarly, the DEGs were primarily involved in oxidative phosphorylation, chemical carcinogenesis-reactive oxygen species, small cell lung cancer, apoptosis, the TNF signaling pathway, and so on. The results of the ssGSEA indicated a statistically significant difference in the apoptosis scores between the control group and the MPs exposure group (*p* < 0.05) (Figure 1C). The analysis of GO enrichment indicated that DEGs had significant enrichment in 1996 BPs, 233 CCs, and 167 MFs (*p* < 0.05). The BP of the DEGs was mainly focused on pathways related to oxidative stress, inflammation and apoptosis. In the CC category, enriched GO terms were predominantly linked to the ribosome. In the MF, DEGs were enriched to the structural constituent of the ribosome, extracellular matrix structural constituent, ubiquitin protein ligase binding, and so on (Figure 1D). The GSEA result indicated activation of the apoptotic pathway in normal lung epithelial cells following exposure to MPs (Figure 1E).

### 3.3. Computational Pharmacology Prediction

**Figure 2:**
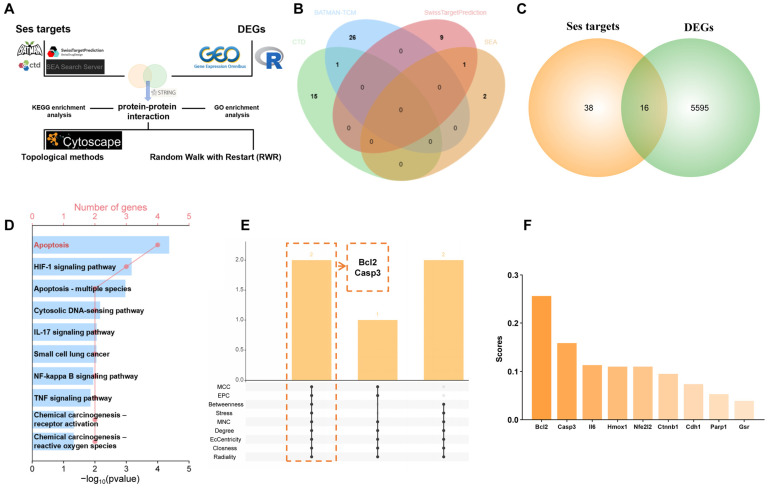
The mechanism of Ses in alleviating MPs-induced lung injury. (**A**) Workflow for network pharmacology analysis. (**B**) Venn diagram showing the numbers of predicted Ses targets. (**C**) Venn diagram showing the intersection of Ses targets with DEGs. (**D**) KEGG enrichment analysis of the intersection targets between Ses targets and DEGs. (**E**) Multiple topological methods were used to identify the core targets in the network. (**F**) Random Walk with Restart based on the PPI network.

The flowchart in Figure 2A represents the network pharmacology analysis process. A total of 54 targets of Ses were retrieved from the CTD, BATMAN-TCM, Swiss Target Prediction, and SEA databases, following the removal of partially duplicate targets (Figure 2B). A total of 16 targets were identified by integrating the targets of Ses with the DEGs (Figure 2C). Subsequent KEGG enrichment analysis of the identified intersection targets revealed significant enrichment in pathways related to apoptosis, the IL-17 signaling pathway, the TNF signaling pathway, and chemical carcinogenesis-reactive oxygen species (Figure 2D). A chord diagram showing the specific genes enriched in apoptosis-related pathways can be found in Figure S3A. GO enrichment analysis of intersection targets revealed significant enrichment of BP terms mainly associated with the response to oxidative stress, response to reactive oxygen species, intrinsic apoptotic signaling pathway, regulation of interleukin-17 production, and lung induction (Figure S3B). All intersection targets were imported into the STRING database to construct the PPI network, which was subsequently analyzed using Cytoscape. The intersection of the three most significant proteins identified in each topological algorithm indicated that Ses may modulate MPs-induced apoptosis through the regulation of Bcl2 and Casp3 (Figure S3C and Figure 2E). In addition, the results of RWR suggested that Bcl2 has a higher score in the PPI network (Figure 2F). Combined, Ses may alleviate MPs-induced lung apoptosis by targeting Bcl2.

### 3.4. Ses Alleviated PS-MPs-Induced Lung Injury in Mice

**Figure 3:**
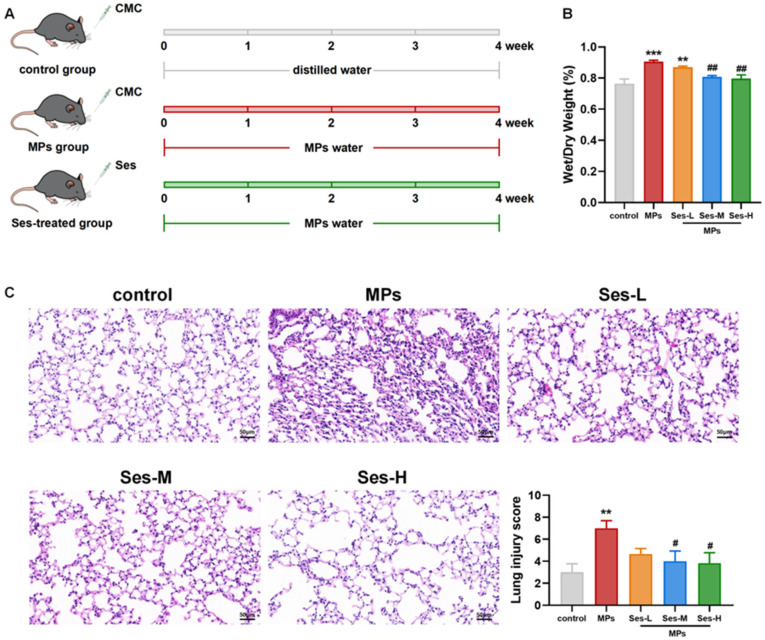
Ses alleviated MPs-induced lung injury in mice. (**A**) Schematic of experimental protocol. (**B**) Ratio of lung wet and dry weight. (**C**) Hematoxylin-eosin (H&E) staining of lung sections and injury score of lung tissues. The data were expressed as the mean ± SEM. All data were obtained from six biological replications. ** *p* < 0.01, versus the control. *** *p* < 0.001, versus the control. # *p* < 0.05, versus the MPs. ## *p* < 0.01, versus the MPs.

The experimental procedure is schematically illustrated in Figure 3A. We evaluated lung water content, which was significantly higher in the PS-MPs-exposed group than in the control (*p* < 0.001). The W/D ratio of the lung was notably reduced in the Ses-treated groups compared with the PS-MPs exposure group (Figure 3B). Lung histopathology indicated that PS-MPs exposure resulted in marked alveolar wall thickening, inflammatory cell infiltration, and lung edema, all of which were remarkably mitigated in Ses-treated groups (Figure 3C). Semi-quantitative analysis of H&E-stained sections revealed that pretreatment with medium and high doses of Ses significantly reduced the lung injury score (*p* < 0.05).

### 3.5. Ses Lightened PS-MPs-Induced Oxidative Stress in Mice

**Figure 4:**
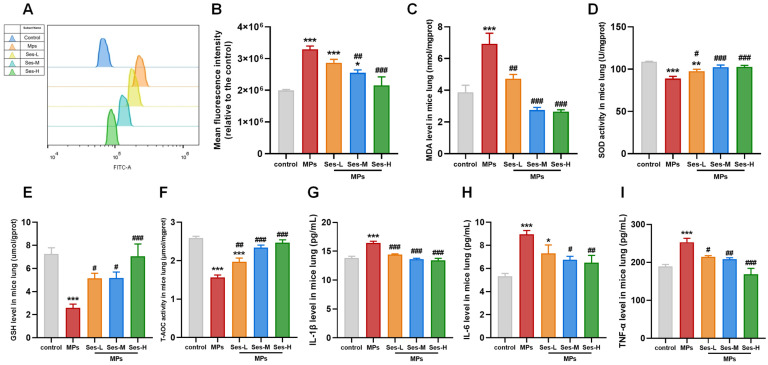
Ses lightened MPs-induced oxidative stress and inflammation in mice. (**A**) Representative fluorescence intensity images of reactive oxygen species (ROS) detected using DCFH-DA and acquired through flow cytometry. (**B**) Flow cytometric analysis of fluorescence intensity. (**C**–**F**) The levels of malondialdehyde (MDA), superoxide dismutase (SOD), glutathione (GSH), and total antioxidant capacity (T-AOC). (**G**–**I**) The levels of interleukin-1β (IL-1β), IL-6, and tumor necrosis factor-α (TNF-α). The data were expressed as the mean ± SEM. All data were obtained from six biological replications. * *p* < 0.05, versus the control. ** *p* < 0.01, versus the control. *** *p* < 0.001, versus the control. # *p* < 0.05, versus the MPs. ## *p* < 0.01, versus the MPs. ### *p* < 0.001, versus the MPs.

Compared with the control group, exposure to PS-MPs significantly elevated the levels of ROS and MDA in lung tissues. However, Ses pretreatment obviously reduced ROS and MDA levels compared with the PS-MPs exposure group (Figure 4A–C). Exposure to PS-MPs resulted in a significant reduction in the activities of SOD, GSH, and T-AOC when compared with the control group. In contrast, pretreatment with Ses markedly enhanced the activities of SOD, GSH, and T-AOC relative to the PS-MPs exposure group (Figure 4D–F).

### 3.6. Ses Diminished PS-MPs-Induced Inflammation Response in Mice

To elucidate the effect of Ses on the inflammatory response, the concentrations of IL-1β, IL-6, and TNF-α in lung tissues were measured. As presented in Figure 4G–I, the levels of IL-1β, IL-6, and TNF-α in lung tissues were significantly increased in the PS-MPs exposure group. Nevertheless, Ses pretreatment significantly reduced this effect. These findings demonstrated that Ses can attenuate the inflammatory response induced by PS-MPs exposure.

### 3.7. Ses Mitigated PS-MPs-Induced Apoptosis in Mice

**Figure 5:**
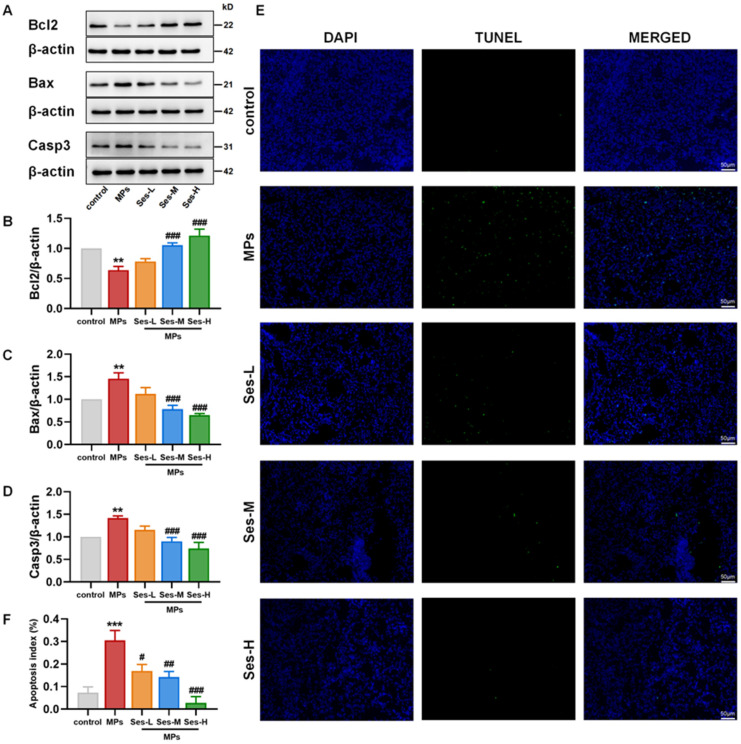
Ses mitigated MPs-induced apoptosis in mice. (**A**) Western blot results for Bcl2, Bax, and Casp3. (**B**–**D**) Quantitative analysis of aforementioned apoptosis-associated proteins. (**E**) TUNEL staining of apoptotic cells in lung tissue sections. (**F**) The ratio of cell apoptosis. The data were expressed as the mean ± SEM. All data were obtained from six biological replications. ** *p* < 0.01, versus the control. *** *p* < 0.001, versus the control. # *p* < 0.05, versus the MPs. ## *p* < 0.01, versus the MPs. ### *p* < 0.001, versus the MPs.

The expression levels of apoptosis-associated proteins, including Bcl2, Bax, and Casp3, were analyzed to investigate whether pretreatment with Ses could mitigate PS-MPs-induced apoptosis in lung tissues. Compared with the control group, PS-MPs exposure significantly reduced Bcl2 expression, while Ses pretreatment substantially restored its levels. Conversely, PS-MPs exposure markedly increased Bax and Casp3 expression, but Ses effectively suppressed their upregulation (Figure 5A–D). Consistent with western blotting results, TUNEL immunofluorescence staining revealed a significantly elevated apoptotic index in the PS-MPs exposure group, which was markedly reduced by Ses pretreatment (Figure 5E,F). These findings indicate that MPs exposure promotes apoptosis in lung tissues, while Ses pretreatment attenuates this effect.

### 3.8. Ses Lightened PS-MPs-Induced Cell Injury in MLE-12 Cells

**Figure 6:**
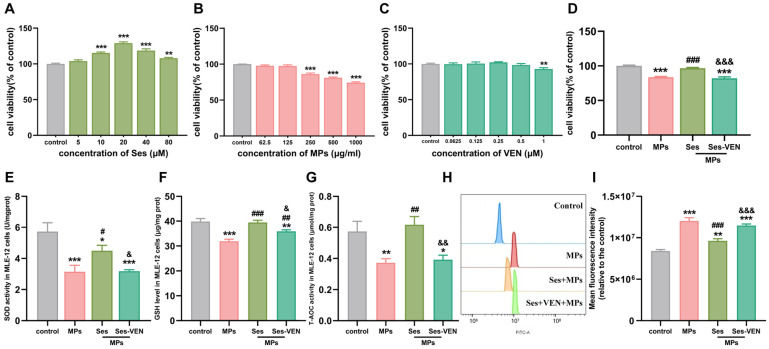
Ses mitigated MPs-induced cell injury in MLE-12 cells. (**A**–**D**) CCK8 was used to test the cell viability. (**E**–**I**) The levels of superoxide dismutase (SOD), glutathione (GSH), total antioxidant capacity (T-AOC), and reactive oxygen species (ROS). The data were expressed as the mean ± SEM. All data were obtained from six biological replications. * *p* < 0.05, versus the control. ** *p* < 0.01, versus the control. *** *p* < 0.001, versus the control. # *p* < 0.05, versus the MPs. ## *p* < 0.01, versus the MPs. ### *p* < 0.001, versus the MPs. & *p* < 0.05, versus the Ses. && *p* < 0.01, versus the Ses. &&& *p* < 0.001, versus the Ses.

To ascertain the optimal concentrations of Ses, PS-MPs, and VEN, MLE-12 cells were treated with different amounts of Ses (0–80 µM), PS-MPs (0–1000 µg/mL), and VEN (0–1 µM) for a duration of 24 h (Figure 6A–C). Cell viability was assessed with the help of the CCK-8 assay kits. Based on these preliminary results, the optimal concentrations were determined to be 20 µM for Ses, 250 µg/mL for PS-MPs, and 1 µM for VEN. To investigate the protective effects of Ses against PS-MPs-induced MLE-12 cell injury, the cells were pretreated with Ses and VEN for 1 h, followed by exposure to PS-MPs for 24 h. The results demonstrated that Ses pretreatment significantly mitigated the reduction in cell proliferation ability induced by PS-MPs exposure. However, the protective effect was diminished when VEN was introduced (Figure 6D and Figure S4A–D). Consistent with previous in vivo results, Ses reversed the reduction in cellular antioxidant capacity induced by PS-MPs, as evidenced by significant increases in SOD, GSH, and T-AOC. Conversely, pretreatment with VEN significantly weakened the protective effect of Ses (Figure 6E–G). In addition, Ses can diminish the production of intracellular ROS triggered by PS-MPs in MLE-12 cells. By contrast, pretreatment with VEN reversed the above changes (Figure 6H,I). Collectively, Ses preconditioning mitigated the cell injury induced by PS-MPs, whereas VEN preconditioning diminished the protective effects of Ses.

### 3.9. Ses Alleviated PS-MPs-Induced Apoptosis via Targeting Bcl2 in MLE-12 Cells

**Figure 7:**
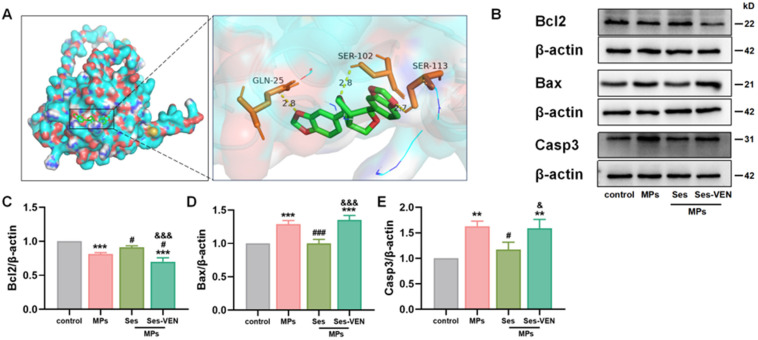
Ses mitigated cellular damage caused by MPs through the modulation of apoptosis. (**A**) Docking results for Ses with Bcl2. (**B**) Apoptosis-related proteins, including Bcl2, Bax, and Casp3, were examined by western blot. (**C**–**E**) Quantitative analyses of Bcl2, Bax, and Casp3. The data were expressed as the mean ± SEM. All data were obtained from six biological replications. ** *p* < 0.01, versus the control. *** *p* < 0.001, versus the control. # *p* < 0.05, versus the MPs. ### *p* < 0.001, versus the MPs. & *p* < 0.05, versus the Ses. &&& *p* < 0.001, versus the Ses.

Molecular docking analyses further confirmed the interaction between Ses and Bcl2 (Figure 7A). The identified binding sites for Ses on Bcl2 included GLN-25, SER-102, and SER-113. The calculated binding energy was −7.7 kcal/mol (lower than −5 kcal/mol), suggesting a robust attraction between the ligand and the protein. Similarly, PS-MPs induced apoptosis in MLE-12 cells, characterized by the downregulation of Bcl2 and the upregulation of Bax and Casp3. However, pretreatment with Ses significantly mitigated these alterations. Notably, pretreatment with VEN diminished the protective efficacy of Ses against PS-MPs-induced apoptosis (Figure 7B–E).

## 4. Discussion

MPs are ubiquitous in the environment, and humans are exposed to MPs through multiple pathways [21]. Current research indicates that ingestion is a primary route of MPs exposure [22]. Furthermore, MPs have been consistently identified throughout all segments of the human lungs, including the upper, middle, and lower areas [22]. Consequently, there is an urgent need for efficient strategies to prevent and mitigate MPs-induced lung injury. Our results revealed that Ses treatment significantly alleviated PS-MPs-induced pulmonary oxidative stress, inflammation, and apoptosis in vitro and in vivo. Importantly, the co-administration of a Bcl2 inhibitor weakened the protective effect of Ses. Mechanistically, integrating computational pharmacology with experimental validation elucidated that Ses exerts a protective role in PS-MPs-induced lung injury by targeting Bcl2.

Transcriptomic approaches have been extensively applied with network pharmacology analyses to elucidate molecular mechanisms [23]. Since MPs accumulate in the lungs through sustained and prolonged ingestion pathways in the human body, understanding the mechanisms of MPs-induced pulmonary injury is essential for preventing and treating lung diseases. The transcriptome data offer insights into the whole-genome dynamics, enabling researchers to elucidate the mechanisms of toxicant action [24]. According to prior studies, the mechanism underlying lung damage induced by MPs is intricate, encompassing the activation of various signaling pathways [13,25,26]. Therefore, the findings remain too fragmented to allow for definitive conclusions. In the study, through comprehensive enrichment analysis of all gene lists and DEGs, we identified a significant enrichment of pathways associated with oxidative stress, inflammation, and apoptosis. The results provide a possible direction for preventing and treating lung injury caused by MPs. Network pharmacology is a mighty method for investigating the possible mechanisms of active ingredients [27]. Numerous studies have shown that Ses exhibits potent anti-inflammatory, antioxidant, and anti-apoptotic properties [28,29,30,31]. In this study, through the integration of network pharmacology approaches, the findings suggest that Ses may mitigate MPs-induced lung injury by modulating the pathways associated with oxidative stress, inflammation, and apoptosis. All the above-mentioned results provide clues and foundations for subsequent experiments.

Oxidative stress plays a pivotal role in driving the pathogenesis and progression of pulmonary diseases [32,33]. Exposure to exogenous MPs represents a critical risk factor in inducing pulmonary oxidative stress [34]. It has been shown that MPs induce ROS generation [35]. The excessive production of ROS surpasses the antioxidant capacity, leading to a redox imbalance [36]. As a biomarker of lipid peroxidation, MDA serves to indicate the degree of cellular damage induced by free radicals [37].

Our findings indicate that PS-MPs exposure leads to elevated levels of ROS and MDA, suggesting that PS-MPs induce oxidative stress in pulmonary tissue. When cells and tissues are exposed to oxidative stress, antioxidant enzymes play vital roles. SOD is a key antioxidant enzyme that can scavenge excess ROS and antagonize the oxidative stress response [38]. Similarly, T-AOC reflects the total non-enzymatic antioxidant capacity against ROS [39]. GSH serves as a powerful antioxidant that mitigates intracellular ROS via a redox reaction, during which it undergoes oxidation to form GSSG [40]. When exposed to excessive oxidative stress, the inordinate production of ROS and MDA and reduced activities of antioxidant enzymes and nonenzymes have been observed. In the present study, PS-MPs significantly increased the levels of ROS and MDA in both in vitro and in vivo models, while Ses decreased the levels of both. Furthermore, PS-MPs caused a significant decrease in the levels of SOD, GSH, and T-AOC. However, Ses pretreatment significantly reversed these effects. These findings demonstrate the protective role of Ses against PS-MPs-induced pulmonary oxidative stress. The Ses intervention may have augmented the metabolic capacity of cells, thereby potentially enhancing their resistance to injury.

It was previously shown that inflammation plays a central part in the progression of MPs-induced pulmonary diseases in both animal models and humans [41,42]. TNF-α is the primary trigger of the inflammatory response, inducing the production and release of pro-inflammatory cytokines such as IL-1β, IL-6, and IL-10, while suppressing the release of anti-inflammatory factors [43]. IL-1β, a member of the interleukin-1 family, serves as a key mediator of inflammatory responses and plays a critical role in regulating cell proliferation and apoptosis during tumorigenesis [44]. IL-6 is a pleiotropic pro-inflammatory cytokine that is essential for the modulation of both innate and adaptive immune responses [45]. It has been demonstrated that inhalation exposure to PS-MPs results in increased levels of TNF-α, IL-1β, and IL-6 in mouse lung tissue [46]. Similar to previous studies, the results showed that PS-MPs significantly elevated the levels of TNF-α, IL-1β, and IL-6 in the lung tissue of mice, whereas Ses pretreatment significantly reduced these pro-inflammatory cytokines. Our findings demonstrate the potent capacity of Ses in ameliorating PS-MPs-induced lung inflammation in mice.

Apoptosis is a key programmed cell death mechanism, regulated by anti-and pro-apoptotic proteins through multiple pathways. Bcl2 and Bax serve as critical regulators of apoptosis, with Bcl2 functioning as an anti-apoptotic factor and Bax acting as a pro-apoptotic factor [47]. In cells, when Bax is highly expressed, Bcl2 and Bax form homodimers via a phosphate diester dehydrogenation bond, thereby promoting cell apoptosis. Conversely, when Bcl2 expression is elevated, the formation of heterodimers between Bcl2 and Bax through hydrogen bonds occurs, effectively inhibiting apoptosis. Casp3 is recognized as a marker of apoptosis, and its elevation may be indicative of apoptotic induction [48,49]. In the mouse model of injury induced by MPs, a series of studies have indicated that apoptosis plays a key role in the multi-organ failure triggered by MPs [50,51,52,53]. In our study, PS-MPs led to a significant reduction in Bcl2 expression and a marked increase in Bax and Casp3 levels, whereas Ses significantly reversed these changes in vitro and in vivo, indicating its anti-apoptotic effect. During the protective effect mediated by Ses, our prediction results suggest that Bcl2 may serve as a target for Ses. In vitro experiments suggested that the Bcl2 inhibitor significantly weakened the antioxidant and anti-apoptotic effects of Ses, further verifying our predictions.

This study demonstrates that the ingestion of PS-MPs can elevate pulmonary oxidative stress, inflammation, and apoptosis levels in vivo, while Ses can exert protective effects. In vitro experiments indicate that Ses may confer a protective effect by targeting Bcl2. Our research underscores the protective potential of Ses as a natural phytochemical; however, extensive validation through clinical trials is necessary before it can be translated into preventive or therapeutic strategies for humans.

Despite these findings, the present study has several limitations. First, the use of a single concentration, size, and type of MPs is insufficient to fully represent the complex real-word human exposure situation. Therefore, future studies should investigate a wider range of exposure variables. Secondly, this study concentrated on investigating the anti-apoptotic properties of Ses. It is recommended that future research also explores other key pathways found to be significantly enriched in this study.

## Data Availability

The raw data supporting the conclusions of this article will be made available by the authors on request.

## Supplementary Materials

The following supporting information can be downloaded at: https://www.mdpi.com/article/10.3390/antiox15030279/s1, Figure S1: The flowchart of the study; Figure S2: The results of transcriptomic data between the control group (CON) and the microplastics-treated lung epithelial cells group (MPs). (A) Principal component analysis (PCA) between CON and MPs. (B) Volcano plot of the differentially expressed genes (DEGs). (C) Heatmap of the DEGs; Figure S3: (A) Chord diagram showing the specific genes enriched in apoptosis-related pathways. (B) GO enrichment analysis of the intersection targets between Ses targets and DEGs. (C) PPI network based on the intersection targets. Figure S4: The proliferation of MLE-12 cells under various intervention conditions.
