# Supplementary material for: Sesamin Protects Against Polystyrene Microplastics-Induced Lung Injury via Attenuating Bcl2-Mediated Apoptosis
Source: Antioxidants (Basel). 2026 Feb 24;15(3):279. doi: 10.3390/antiox15030279 (PMC13023509; doi:10.3390/antiox15030279)
Supplement: Supplementary file 1 [file antioxidants-15-00279-s001.zip › antioxidants-4128526-supplementary.pdf]

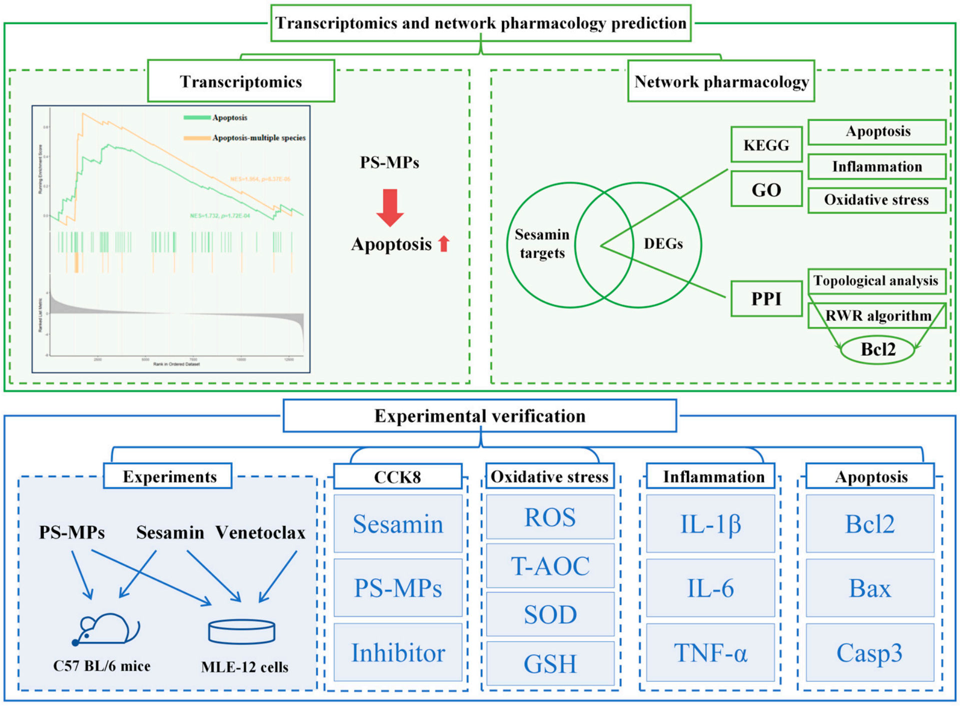

Fig. S1 The flowchart of the study.

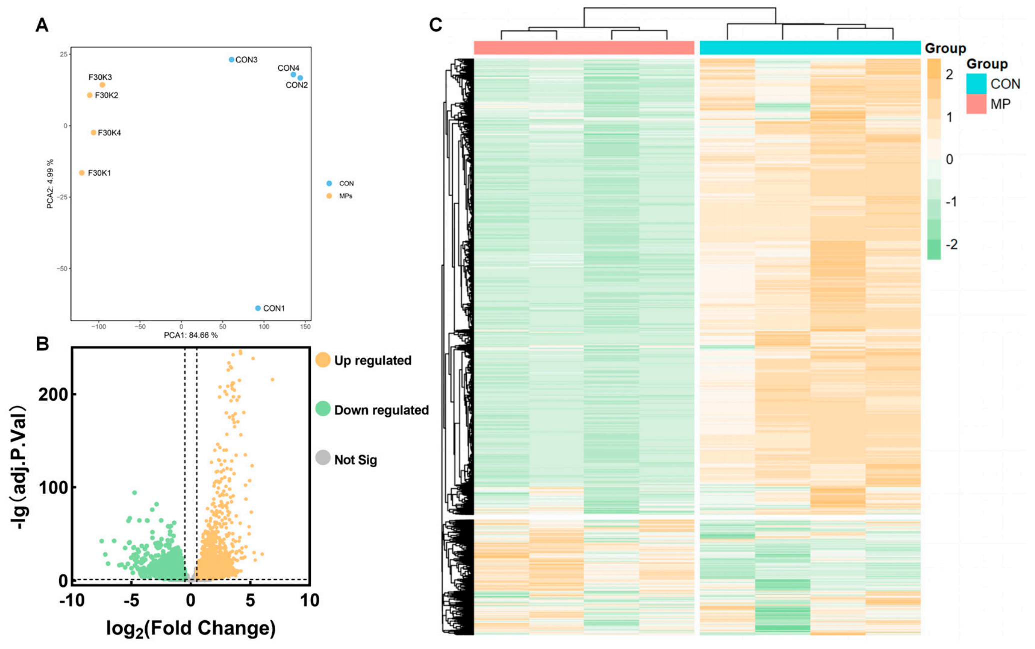

Fig. S2 The results of transcriptomic data between the control group (CON) and the microplastics-treated lung epithelial cells group (MPs). **A** Principal component analysis (PCA) between CON and MPs. **B** Volcano plot of the differentially expressed genes (DEGs). **C** Heatmap of the DEGs.

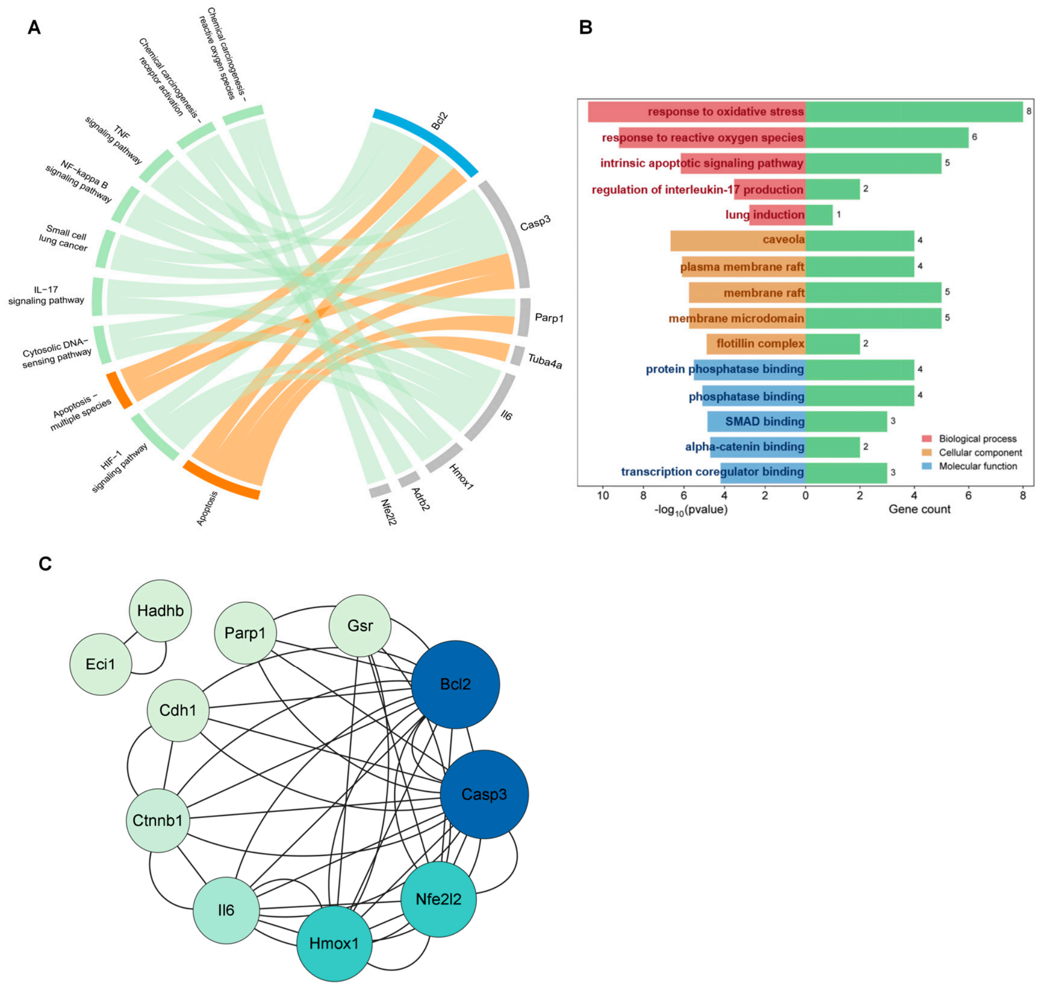

Fig. S3 A Chord diagram showing the specific genes enriched in apoptosis-related pathways. **B** GO enrichment analysis of the intersection targets between Ses targets and DEGs. **C** PPI network based on the intersection targets.

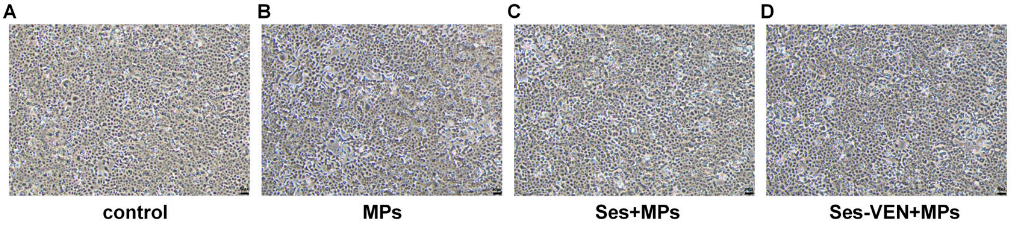

Fig. S4 The proliferation of MLE-12 cells under various intervention conditions.
